# Indents

**Published:** 1905-01-15

**Authors:** 


### INDENTS.

**☞ Brief Articles of Technical or Scientific Value will receive notice and comment. Contributions invited.**

**Dummies.** All dummies should be completed in individual form before permanently assembling. This minimizes the danger of fracturing facings because of the contraction of a large mass of solder. It also facilitates construction, soldering and final finishing.—Goslee, in *Items*.

**Argoral.** I have been particularly interested of late years in the rather surprising results I have accomplished by the use of a robin hue salt of silver, and I would like you gentlemen to try it. Being a general surgeon with a leaning towards the mouth, I have had no great opportunity of trying it in pyorrhea alveolaris, but it has accomplished results in suppuration elsewhere which have been marvelous.

The chemical I refer to is argoral, and if I could have but one antiseptic that is the one I would have. It is better than any of the other salts of silver, being twenty-five percent in strength as against eight percent, at the most, of the other salts. It is very freely soluable, and though it is marvelously active in attacking microbes, it is equally marvelous in being so unirritating. I have made a saturated solution of it in water and applied it to my own eyelids, when I have had congestion of the eyelids from reading too much, and without irritation. Among men who are specialists in other lines of work, gonorrhea, for instance, the genital urinaries, it is admitted the best chemical in its power to attack microbes. It is also non-poisonous; a considerable amount could be swallowed without poisonous results. Therefore, if you made a saturated solution of that in water, which would look almost as black as ink, and applied it to the gums by syringing, good results might be expected. The patients would then be given a small supply of it, not a prescription but the medicine itself, and directed to apply it, say twice a day, with a tiny swab on a tooth pick to the gingival mucus membrane where it seemed to need it, as it would then extend by capillary attraction between the teeth and gums. If it acts as well there as elsewhere, you will find a remedy which is by no means contemptible.—*Items of Interest.*

**Infection from Impacted Root.** A gentleman upward of sixty, who called upon me and said that two and a half years previously he had a badly-inflamed jaw and face and was unable to open his mouth. He was fed through a tube. He went to a physician, who stated that he had a malignant growth and suggested that he go to a specialist. The specialist concurred in the opinion and sent him to a surgeon for operation. The tissues were laid open, the bone scraped, and the man told that he would recover. From that time the man had a sinus discharging on the border of the jaw. Fortunately he had a heavy growth of whiskers which covered it, but he was obliged to carry absorbent cotton to keep it in a respectable condition. The history was the familiar one of alveolar abscess, although there was nothing on the inside of the mouth to indicate one. There was no sign of an embedded root, although I suspected one, and suggested that the man have a skiagraph taken. The first one taken of the entire jaw showed a faint outline of a root. That was not satisfactory, and I had a second one taken, using a film on the side of the border of the jaw, and that showed a root plainly. I made an application of cocain and cut down upon it, but it was too firmly embedded to be removed by the forceps. I used a surgical burr, took the root out, and passed a probe from the opening on the outside of the face through the tissues and found that it came out through an opening in the bone. This was washed out with antiseptics. There was no discharge the next day, and on the second day the opening on the face had closed up. I opened it again, fearing that it had closed too quickly, but from the third day the wound has remained closed and there has been no discharge. I saw the patient this afternoon. The inside of the mouth presents an entirely healthy appearance and the wound is entirely healed. For this credit is due to the X-ray diagnosis.— Dr. Gaylord, in *International.*

**Method of Administering Somnoforme.** Employ always a mask or inhaler in preference to a handkerchief or waterproof cone with which it was originally applied. The inhaler will permit not only the exact measurement of the dose employed, but also the rapid induction of anesthesia by the total exclusion of air, a factor of great importance. After seating the patient with his head in line with the body, explain to him that he is to make deep inhalations, that the liquid has a slight irritating odor, and that it will produce a quiet and agreeable sleep, if he thinks of something pleasant. The pneumatic pad of the inhaler having been inflated and tried on the patient's face pour the somnoforme from the bottle into the chamber of the inhaler in a dose of five cubic centimeters or the contents of a capsule, such as they are sold by the manufacturer, close very rapidly the chamber of the inhaler and instantly apply the face-piece to the patient.

Generally in about twenty seconds the action of the agent will commence and the signs of complete anesthesia will be seen by the cessation of the occular movements, drooping of the eyelids, dilating of the pupils, complete relaxation occasional rigity of the arms, and loss of corneal reflex. The period of induction is completed in from 30 to 45 seconds and the anesthesia last from sixty to ninety seconds. The pulse slightly increases in frequency and tension and the color of the face remains completely normal without traces of the cyanosis that appears when nitrous oxide is employed When the patient commences to recover, analgesia persists during some seconds, allowing a little more time to operate with the patient in a semi-conscious state. In four or five minutes the patients completely recover.

In conclusion, I consider somnoforme the most valuable general anesthetic in dental practice—by the rapidity of its induction (thirty seconds) and the length of its duration (fifty seconds) by the possibility of administering it to all patients and without especial preparation, by its pleasant effects and by its safety, demonstrated not only by the investigations on its action on the nervous centers, but also by a clean record of over 300,000 cases.—*Items.*

**Ratio of Sexes Treated.** Through the kindness of a number of our brothers in the profession, we are by their records able to show the percentage of work done for the two sexes in a period extending over a number of years. The first record submitted was from a practice almost exclusively operative, and shows that 39 percent was for men and 61 percent for women, a difference of 22 percent. The next is a record of a general practice, and shows five men to every eight women, or 38½ percent of men and 61½ percent of women, a difference of 23 percent.

Then we have a record of 1,514 patients, covering a period of five years in one of the oldest general practices in the city, which shows that 591 of the patients were men and 937 women, or practically 39 percent and 61 percent, again showing a difference of 22 percent.

The next is exclusively a prosthetic practice, embracing some 904 patients. We find that 710 are women and 194 men, or about 78½ percent women and 21½ percent men—a difference of 57 percent. But the actual work completed, based upon the charges, shows that but 64 percent of the work done was for women and 36 percent for men—a difference of only 28 percent, or a gain for men of about 50 percent of the difference in cost of work completed, as against the number having work done.

This difference may be explained by two facts: First, where caries exists in man he is less careful about having his teeth cared for, and if troubled, resorts to extraction rather than submit to filling. Second, when he is having work done, he wants the very best that his income will permit him to obtain, while the woman saves all teeth posible, and when compelled to consult our prosthetic brother, she has just as little to be done in his line as possible; and, inasmuch as "John" is to pay the bill, she wants it done just as cheaply as consistent with good, serviceable work.

In our own practice, exclusive of the surgical side, considering simply the operative part of it, for four years and a half we find the percentage runs 63 women to 37 men—a difference of 26 percent.

The investigation of the records of some 31,863 extractions made prior to and during our practice in this city, reveals the fact that about 77 percent were for women and 23 percent for men—a difference of 54 percent. The examination of our records made since locating in the city h ows the ratio to be about seven teeth for women to three or men, or a difference of 40 percent. Thus we find that when all branches of the dental art are considered, woman demands from 22 percent to 57 percent more of our work than man; or to be more explicit, taking the general average we find woman demands about 33⅓ more attention at our hands than does man.—W. G. Ebersole, in *Items of Interest.*

**Blue Light as an Anesthetic.** "The other day the Geneva correspondent of the *London Daily Mail* personally tested a new system, by which the glamor of romance, the joys of the lotus, are lent to the extraction of teeth. Dr. Camille Redard, a Swiss dentist, has discovered the method whose efficacy, nay, whose whole philosophy, is based on the employment of blue light. (This is probably only a new form of the blue window pane cure of thirty-five years ago. Some of these panes may yet be seen in Boston windows.)

"Having placed the correspondent in a recumbent position, the doctor impressed upon him the great importance of faith in the new process, and then commanded him to gaze steadily at an ordinary sixteen-candle power electric light, with a blue glass bulb, fixed within eight inches of his eyes. Behind the lamp was a reflector. After covering his patient with a large blue cloth the doctor left the room.

" 'I found myself gazing at the dazzling blue light, which gradually seemed to penetrate my eyes and pass out of the back of my head,' writes the correspondent.

" 'At first I felt a slight burning sensation, which in turn gave way to one of coolness around the eyeballs. This feeling passed, and I felt nothing more out of the ordinary except that a sensation of rest came over me, and my hands, which were trembling slightly before, were perfectly still now. My senses never left me and I plainly heard the doctor entering the room.

" 'He took away the blue cloth rapidly, placed the blue electric light farther away, tilted the chair up slightly, and signed to me with the instrument to open my mouth.

" 'I felt the instrument grasp the tooth and watched the doctor pull. The next instant I saw the molar before me, not having felt the slightest pain.

" ' "Any pain?" asked Dr. Redard, and added quickly, "I should not ask that question, because I saw by your face that you felt nothing." And it was true.

" 'There were no after-effects, and I found myself seated in the waiting room soon after, chatting pleasantly with the doctor.

" ' "During my personal experience," said the doctor, "I found that the blue light has a soothing effect on the optic nerve, and I put the discovery to practical use. I obtained also nearly the same effect with the green light, but I had more failures, and I discarded it for the present method.

" ' "I am still continuing my experiments with the different colors," he added, "which I am convinced, when their real properties are fathomed, will produce a revolution not only in dnetistry, but in therapeutics, in which we are so backward." ' Ocean steamers may one day be propelled by the simple use of red."

